# Feasibility study and reference values of FibroScan 502 with M probe in healthy preschool children aged 5 years

**DOI:** 10.1186/s12887-019-1487-6

**Published:** 2019-04-24

**Authors:** Jing Zeng, Xi Zhang, Chao Sun, Qin Pan, Wen-Yi Lu, Qian Chen, Li-Su Huang, Jian-Gao Fan

**Affiliations:** 1 Center for Fatty Liver, Department of Gastroenterology, Xinhua Hospital Affiliated to Shanghai Jiao Tong University School of Medicine, Shanghai, 200092 China; 2 Department of Pediatrics, Xin Hua Hospital Affiliated to Shanghai Jiao Tong University School of Medicine, Shanghai, 200092 China; 3 Department of Clinical Nutrition, Xin Hua Hospital Affiliated to Shanghai Jiao Tong University School of Medicine, Shanghai, 200092 China; 4 Ministry of Education-Shanghai Key Laboratory of Children’s Environmental Health, Xinhua Hospital, Shanghai Jiao Tong University School of Medicine, Shanghai, 200092 China; 5 Center for Fatty Liver, Department of Gastroenterology, Xinhua Hospital Affiliated to Shanghai Jiao Tong University School of Medicine, Shanghai Key Lab of Pediatric Gastroenterology and Nutrition, 1665 Kongjiang Road, Shanghai, 200092 China

**Keywords:** Transient elastography, Reference values, Liver stiffness measurement, Controlled attenuation parameter, Children

## Abstract

**Background:**

Transient elastography (TE) using FibroScan with M probe has been widely used in adults for controlled attenuation parameter (CAP) and liver stiffness measurement (LSM). In this study, we aimed to assess the feasibility of this approach and reference values of CAP and LSM in healthy preschool children aged 5 years.

**Methods:**

FibroScan-502 with M probe (Echosens, Paris, France) and bioelectrical impedance analysis (InBody 720, Biospace, South Korea) were prospectively conducted in healthy children aged 5 years from the Shanghai Prenatal Cohort Study. Linear regression models and piece-wise linear regression models were used to explore the factors associated with CAP and LSM.

**Results:**

The success rate of a valid TE measurement was 96.5% in 452 healthy preschool children aged 5 years, and 436 children with 236 boys were included for further study. The median, inter quartile range (IQR) and the 5th–95th percentiles of CAP values were 171.50, 162.07–188.13 and 154.21–214.53 dB/m, respectively. The median, mean ± standard deviation and the 5th–95th percentiles of LSM were 3.20, 3.28 ± 0.86 and 2.00–4.78 kPa, respectively. In multivariate linear regression analyses, the CAP but not the LSM value was significantly positively correlated with such anthropometric index as body weight, body mass index, waist circumference, body fat content and body fat percentage.

**Conclusions:**

FibroScan-502 with M-probe can be used to measure CAP and LSM in preschool children aged 5 years. The 95th percentiles of CAP values and LSM were 214.53 dB/m and 4.78 kPa, respectively. Further study should be performed to explore the cut-off values of CAP and LSM for diagnosis of hepatic steatosis and fibrosis in children.

## Background

Liver biopsy is traditionally considered the gold standard for diagnosis of hepatic steatosis and fibrosis, but its use as a screening tool in apparently healthy individuals is limited because of its costly and invasive nature, sample errors, and intraobserver and interobserver variability [1–3]. Recently, some novel noninvasive techniques for assessment of liver fat and fibrosis have been developed. Transient elastography (TE) is one of these new techniques based on inducing a shear wave to the liver and measuring the velocity of the wave. The device (FibroScan-502, Echosens, Paris, France) was developed using the TE technique, and controlled attenuation parameter (CAP) and liver stiffness measurement (LSM) can be obtained simultaneously by the device in a rapid, noninvasive, reproducible and painless way.

FibroScan-502 with M-probe has been widely used in adults with chronic liver diseases and during health examinations, and the CAP and LSM values were found to be closely related to liver biopsy-proven steatosis and fibrosis, respectively [4–9]. However, FibroScan-502 with M-probe has seldom been used in assessment of liver fat and fibrosis in pediatric individuals with liver diseases, and the reference values of CAP and LSM have not yet been studied well in a large sample of preschool healthy children [10–14]. Therefore, this study explored the feasibility of this technique with M probeto determine the normal range and factors associated with CAP and LSM values in preschool children aged 5 years from the Shanghai Prenatal Cohort, Shanghai, China.

## Methods

### Study population

The individuals included in the study were 5-year-old (58–62 months) children from the Shanghai Prenatal Cohort Study, which was an ongoing perspective birth cohort that enrolled 1043 Han maternal-child pairs between January 2012 and December 2013 at Xinhua Hospital and International Peace Maternity and Child Hospital in Shanghai. All children in the cohort received annual follow-ups with some medical examinations. The exclusion criteria for the study population were as follows: (i) the diagnosis of any type of liver disease; (ii) extraliver diseases and/or therapeutic drugs that might affect liver fat and liver function tests; (iii) body weight over two standard deviations (SD) of the average weight for healthy children of the same sex, age or height; (iiii) measurement failure of FibroScan-502 with M probe. Ethics approval was obtained by the Ethics Committees of both Xinhua Hospital affiliated with the Shanghai Jiao Tong University School of Medicine and the International Maternal and Children Care Hospital. The parents of all the participating children were required to give informed consent to the study and sign the written documents.

### Clinical and laboratory data collection

All participating children underwent annual medical examination at the health examination center in Xinhua Hospital. Stadiometers (Seca 416 Infantmeter, USA) were used to measure height to the nearest 0.1 cm. Digital scales (Detector 6745 Baby Scale, USA) were used to measure body weight to the nearest 0.1 kg. Fat mass and the percent of body fat were obtained through bioelectrical impedance analysis (InBody 720, Biospace, South Korea). Participant characteristics and anthropometric indices, including age, sex, body weight, height, waist circumference, body mass index (BMI), fat mass and the percent of body fat were obtained. Blood samples were collected in the morning after at least 12 h of fasting, and serum levels of alanine amino transferase (ALT), gamma-glutamyl transferase (GGT), alkaline phosphatase (AKP), triglyceride (TG), high-density lipoprotein cholesterol (HDL-C) and low-density lipoprotein cholesterol (LDL-C) were measured with a Hitachi 7600 series automatic analyzer (Hitachi, Tokyo, Japan).

### Assessment of liver fat and fibrosis by transient elastography

Following a fast of at least 6 h, all participants underwent FibroScan-502 with M-probe (3.5 MHz) examination (Echosens, Paris, France) by the same physician (JZ). The device estimates liver stiffness in kilopascal (kPa) and liver steatosis in decibel/meter (dB/m). CAP in dB/m and LSM in kPa were obtained simultaneously by each examination. A TE examination was considered successful when 10 valid measurements with a success rate of at least 60% were conducted and the interquartile range (IQR) was less than 30% of the median LSM value [15]. Subjects with unsuccessful examinations were excluded from the analyses.

### Statistical analysis

Continuous variables were expressed as means ± SD for a normal distribution and median ± IQR for a skewed distribution. The t-test and χ^2^ test were used to test intergroup differences between different genders for continuous and categorical variables, respectively. Correlations between the CAP and LSM values and clinical parameters were tested using Spearman’s correlation analysis. The potential influencing factors associated with CAP and LSM values were analyzed using a univariate linear regression model. Multivariate linear regression models and piecewise linear regression models were applied to examine the nonlinear relationships between CA*P* values and covariates. Statistical analyses were performed using R (http://www.R-project.org). Statistical significance was indicated by two-sided *P* values < 0.05.

## Results

### Participant characteristics

**Fig. 1:**
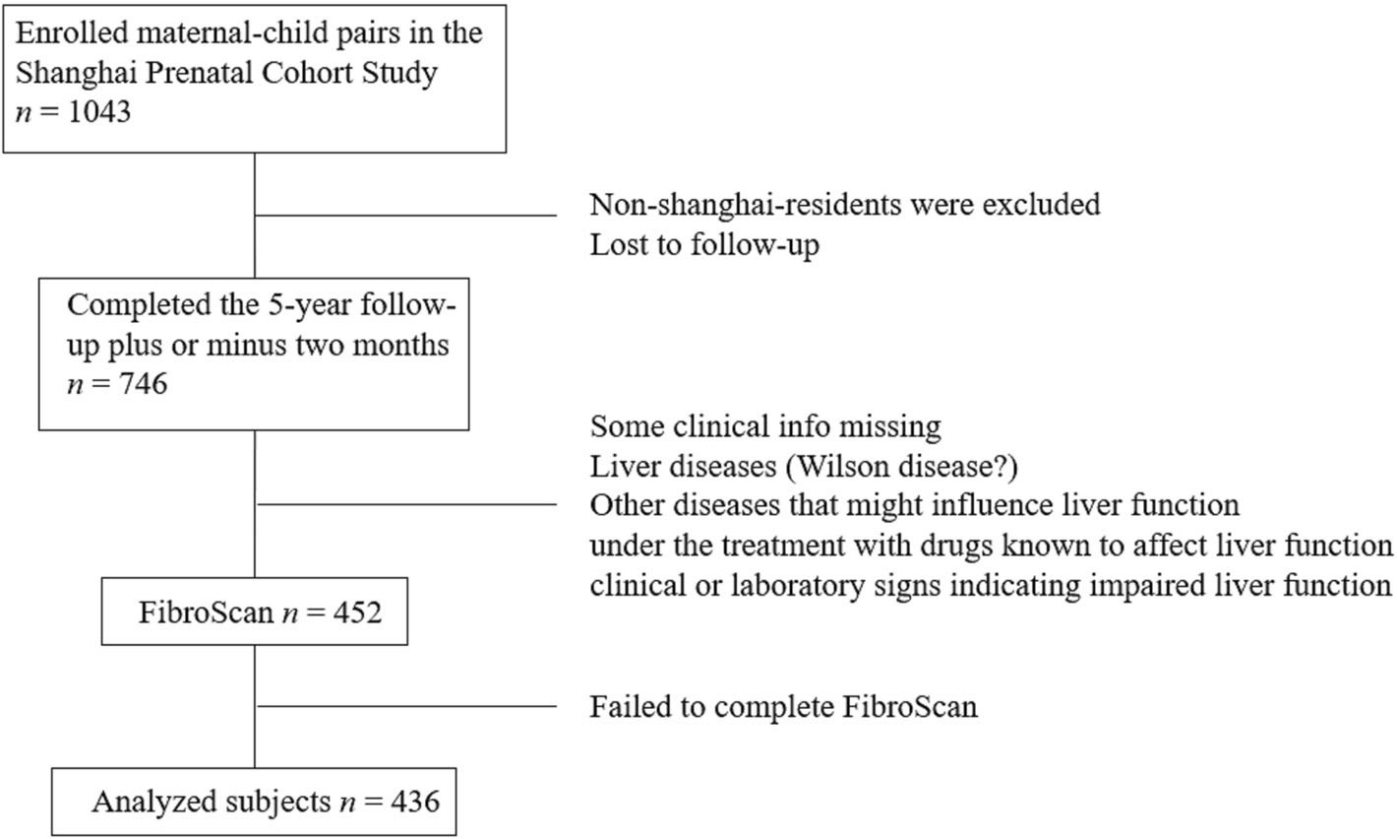
Flow diagram of participants included in this study

From the Shanghai Prenatal Cohort Study, 746 maternal-child pairs prospectively received a 5-year (58–62 months) follow-up. Of these individuals, 452 participants were enrolled into the TE study, and the success rate of valid FibroScan measurements was 96.5% (436/452) with 16 children excluded (10 because of intercostal space that were too narrow, 4 because of moving, and the remaining cases because of unknown reasons). Finally, 436 children aged 5 years old (58–62 months) were included in the analysis (Fig. 1). As shown in Table 1, all enrolled participants had normal anthropometric indices and normal ranges of serum ALT, AKP, GGT, TG and HDL-C. Only body weight, BMI, and waist circumference in boys were significantly higher than those in girls, whereas the level of LDL-C was lower in boys than in girls (all *P* < 0.05).

**Table 1:** Characteristics of 436 healthy children aged 5 years

Characteristics	Total (*n* = 436)	Boys (*n* = 236)	Girls (*n* = 200)	*P* value
Height (cm)	111.0 ± 4.5	111.5 ± 4.5	110.6 ± 4.5	0.053
Height Z score	−1.9 ± 6.8	−1.5 ± 6.1	−2.0 ± 6.7	0.057
Body weight (kg)	19.5 ± 3.1	20.0 ± 3.3	18.9 ± 2.8	0.000
Body weight Z score	− 0.5 ± 2.0	− 0.3 ± 1.9	− 0.6 ± 1.9	0.027
BMI (kg/m2)	15.7 ± 1.8	16.0 ± 1.9	15.4 ± 1.5	0.000
BMI Z score	−0.6 ± 2.7	−0.4 ± 2.5	−0.6 ± 2.6	0.100
Waist circumference (cm)	52.6 ± 4.6	53.2 ± 4.8	51.9 ± 4.4	0.007
Fat mass content (kg)	3.60 ± 1.96	3.62 ± 2.02	3.59 ± 1.93	0.854
Percent of body fat (%)	17.7 ± 6.9	17.3 ± 6.8	18.2 ± 7.0	0.204
ALT (U/L)	11(10, 13)	11(10, 13)	11(9, 13)	0.190
AKP (U/L)	215 (181, 245)	217 (182, 248)	213 (180, 242)	0.647
GGT (U/L)	10 (9, 12)	10 (9, 12)	10 (9, 12)	0.895
TG (mmol/L)	0.95 (0.73, 1.26)	0.95(0.71, 1.32)	0.96 (0.75, 1.23)	0.201
LDL-C (mmol/L)	2.40 ± 0.65	2.30 ± 0.64	2.50 ± 0.65	0.004
HDL-C (mmol/L)	1.55 ± 0.34	1.52 ± 0.35	1.58 ± 0.32	0.114

*P*-values are for chi-square or Kruskal-Wallis tests between boys and girls. Means ± SDs were calculated for variables of normal distribution; median and interquartile range (IQR) are shown for variables of skewed distribution

*BMI* body mass index, *ALT* alanine amino transferase, *AKP* alkaline phosphatase, *GGT* gamma-glutamyl transferase, *TG* triglyceride, *LDL-C* low-density lipoprotein cholesterol, *HDL-C* high-density lipoprotein cholesterol

### Normal range of CAP and LSM values in preschool children aged 5 years

**Fig. 2:**
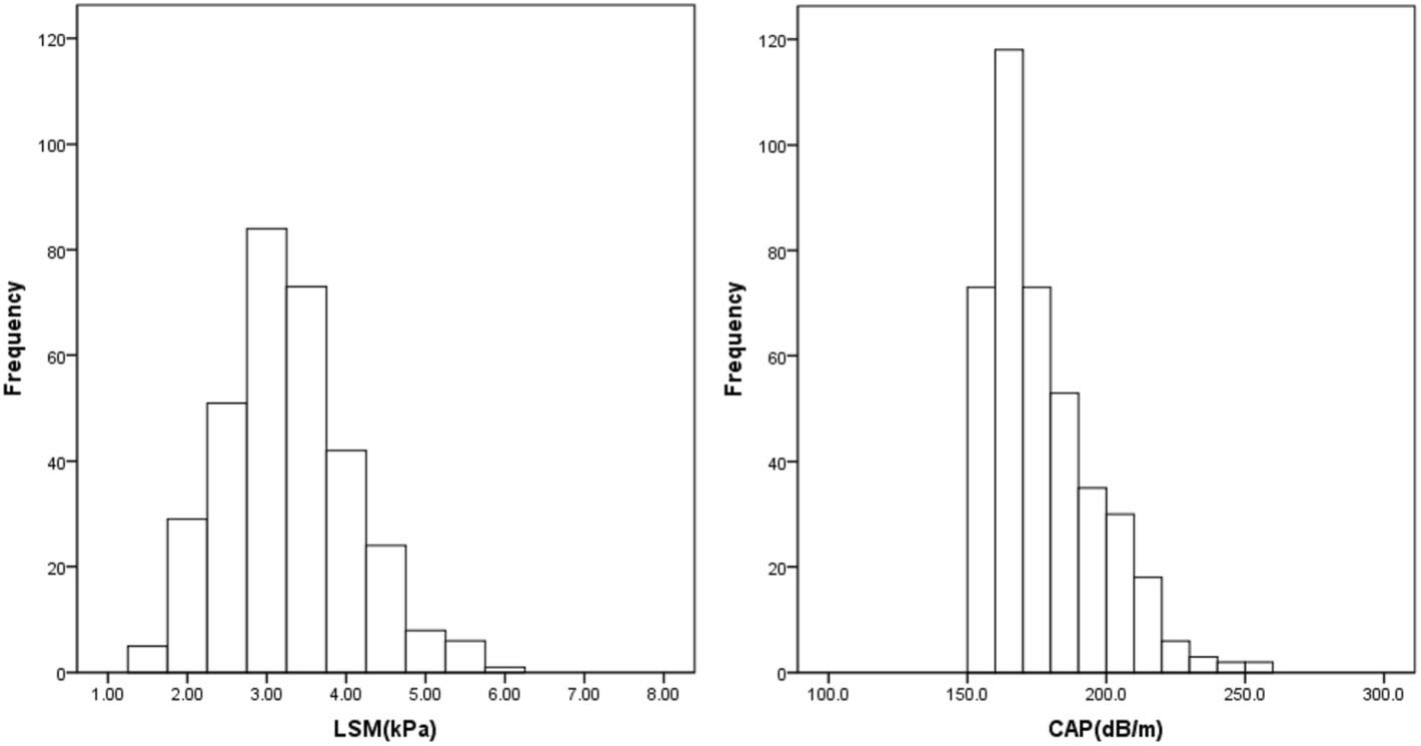
The frequency distribution histogram of FibroScan measurements in 436 preschool children aged 5 years. LSM, liver stiffness measurement; CAP, controlled attenuation parameter

Valid CAP and LSM values were available from 436 children who underwent successful examination of FibroScan-502 with M probe. The frequency distribution and reference ranges of the CAP and LSM values in 436 children aged 5 years are presented in Fig. 2 and Table 2, and the CAP and LSM values were very similar between boys and girls. No variables listed in Table 1 were found to be associated with LSM values in the 436 participating children (data not shown).

**Table 2:** LSM and CA*P* values in 436 healthy children aged 5 years

	Total (*n* = 436)	Boys (*n* = 236)	Girls (*n* = 200)
Median of LSM (kPa)	3.20	3.20	3.20
Mean ± SD of LSM (kPa)	3.28 ± 0.86	3.29 ± 0.89	3.28 ± 0.84
5th - 95th percentile values of LSM (kPa)	2.00–4.78	1.90–4.90	2.03–4.58
Median of CAP (dB/m)	171.5	171.4	171.7
Interquartile range of CAP(dB/m)	162.1–188.1	162.2–187.0	162.0–191.5
5th - 95th percentile values of CAP (dB/m)	154.2–214.5	152.9–214.9	155.3–214.8

*LSM* liver stiffness measurement, *CAP* controlled attenuation parameter. All *P* value > 0.05 between boys and girls

### Factors associated with CAP values

In univariate and multivariate linear regression analyses, the CAP values were significantly positively correlated with body weight, BMI, waist circumference, fat mass and percent of body fat (data shown in Table 3) in 436 healthy children.

**Table 3:** Univariate and multivariate analyses of factors related to CAP (dB/m) in 436 healthy children aged 5 years

	Univariate analysis		Multivariate analysis^a^	
	r-value	*P*-values	CAP ß (95% CI)	*P*-values
Height (cm)	0.112	0.026	0.3 (−0.2, 0.7)	0.072
Body weight (kg)	0.145	0.004	1.0 (0.5, 1.5)	< 0.001
BMI (kg/m^2^)	0.114	0.023	1.9 (1.0, 2.8)	< 0.001
Waist circumference (cm)	0.178	0.000	0.7 (0.4, 1.0)	< 0.001
Fat mass content (kg)	0.125	0.005	1.4 (0.5, 2.2)	0.001
Percent of body fat (%)	0.099	0.027	0.3 (0.1, 0.5)	0.040
ALT (U/L)	0.047	0.377	–	–
AKP (U/L)	0.008	0.887	–	–
GGT (U/L)	−0.042	0.422	–	–
TG (mmol/L)	0.116	0.027	1.6 (−1.8, 5.0)	0.365
LDL-C (mmol/L)	−0.121	0.021	0.3 (−1.9, 2.1)	0.345
HDL-C (mmol/L)	−0.095	0.072	–	–

Abbreviations: *CAP* controlled attenuation parameter, *BMI* body mass index, *ALT* alanine amino transferase, *AKP* alkaline phosphatase, *GGT* gamma-glutamyl transferase, *TG* triglyceride, *LDL-C* low-density lipoprotein cholesterol, *HDL-C* high-density lipoprotein cholesterol

^a^Factors < 0.05 in the univariate analysis were included in the multivariate analysis

**Fig. 3:**
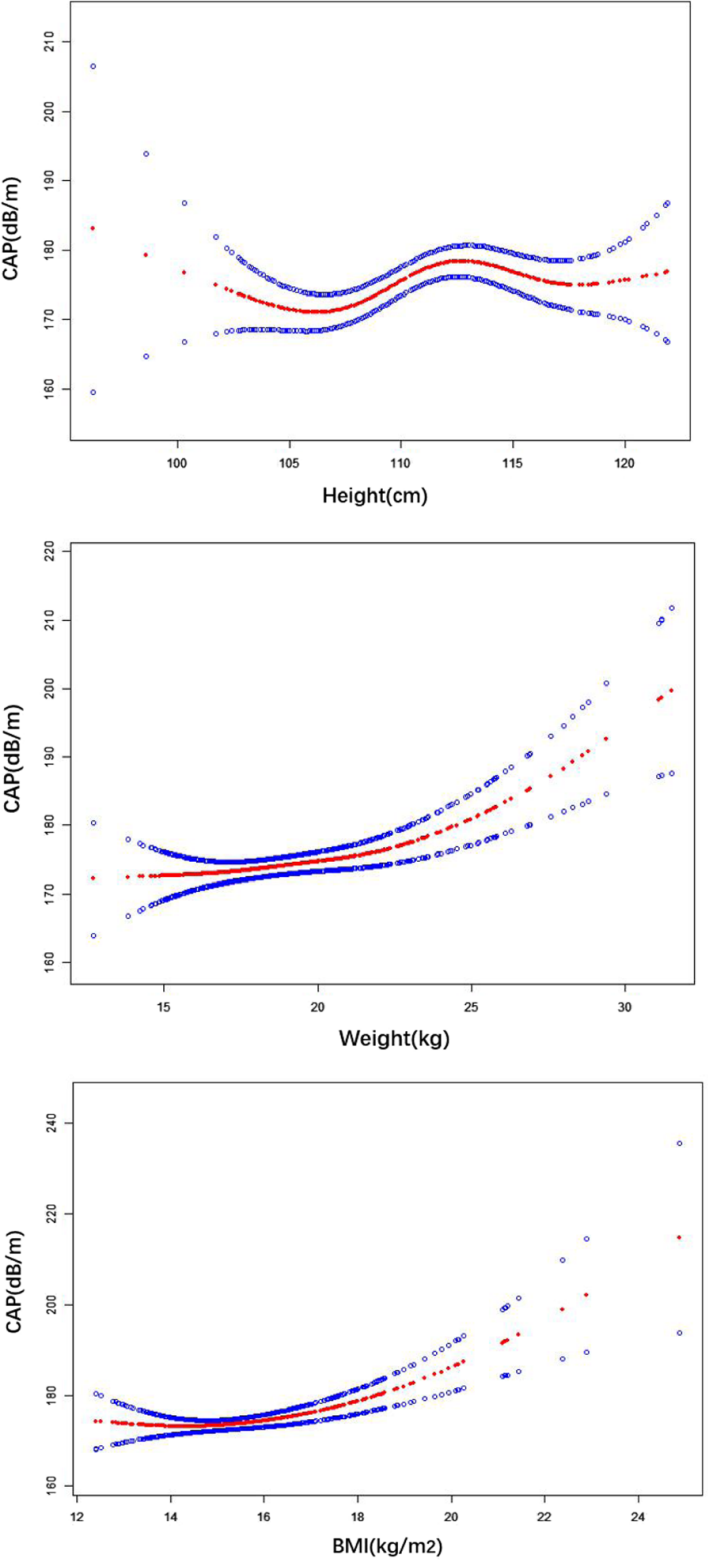
The nonlinear associations between anthropometric measurements (height, weight, BMI) and CA*P* values in children aged 5 years. Piecewise linear model results indicated no significant regression between CAP values and height, a significant positive association between CAP values and weight, and a significant positive relationship between CAP values and BMI in the children

Additionally, the piece-wise linear regression model results indicated significant nonlinear relationships between CAP values and the children’s anthropometric measurements (body weight and BMI). (Fig. 3, Table 4) .

**Table 4:** Piece-wise linear analyses of factors related to CAP(dB/m)

	Height		WEIGHT		BMI	
CAP	ß (95%CI)	*P* value	ß (95%CI)	P value	ß (95%CI)	*P* value
Turning point(TP)	105.9		22.1		17.84	
Lower than TP	−1.22 (−3.32, 0.89)	0.2574	0.18 (−0.65, 1.01)	0.6761	0.28 (−1.07, 1.62)	0.6859
Higher than TP	0.61 (0.16, 1.06)	0.0087	2.81 (1.44, 4.19)	< 0.0001	6.20 (3.30, 9.11)	< 0.0001

Abbreviations: *CAP* controlled attenuation parameter, *BMI* body mass index

## Discussion

This study is by far the largest survey using FibroScan-502 with M probe in preschool children who underwent healthy checkups each year from birth. The mean values of LSM, median values of CAP and the ranges of LSM and CAP values in a total of 436 healthy children at 5 years old were obtained in the study.

After the development of TE for more than ten years, FibroScan has been applied in many studies to evaluate adult liver disease [4–9]. Currently, FibroScan is recommended in many countries and areas as a routine diagnostic for liver fibrosis [16, 17]. CAP is now increasingly used to compensate for the role of liver biopsy in the evaluation of hepatic steatosis [7–9]. In recent years, FibroScan has also been applied in studies focusing on the diagnosis and follow-up of pediatric liver diseases, mainly concentrating on biliary atresia [10, 18] and chronic liver disease [11–13]. In addition, many studies on the normal ranges of LSM and CAP in a large sample of healthy children with different ages have already been undertaken. Cho et al. found that the mean value of LSM measured by FibroScan in healthy infants was (3.9 ± 0.9) kPa [15]. Goldschmidt et al. also found that the mean value of LSM was 4.5 (2.5–8.9) kPa by evaluating 527 healthy children (age 0.1–17.8 years, the average age 6.0 years) without any basic diseases [19]. Tokuhara et al. determined the LSM and CAP values in 123 children (age 1–18 years) using FibroScan, and the final results showed that there were significant differences of the mean values of LSM between each age group (*p* = 0.001), with 3.4 (2.3–4.6 kPa) in children aged between 1 and 5 years, 3.8 (2.5–6.1) kPa in children aged between 6 and 11 years and 4.1 (3.3–7.9) kPa in children aged between 12 and 18 years, respectively, and the mean CAP value was 183 (112–242) dB/m which did not change with age [20]. They thus concluded that the LSM values increased with age. Engelmann et al. also reported that the LSM values increased with age [21]. However, those studies were focused on children with liver diseases or healthy children at different ages, and most studies only measured LSM values without CAP values. As LSM values increased with age, we believe that age could lead to variations in the diagnostic accuracy of TE in a screening setting for hepatic fibrosis and steatosis in apparently healthy individuals [22, 23]. As CAP values were also important for children to identify fatty liver diseases, the normal LSM and CAP values in children at every age should be determined distinctively. Our study used FibroScan to evaluate 436 children (age 58–62 months) of the Shanghai Prenatal Cohort who underwent healthy checkups between August to November 2017. The mean LSM and median CAP values were 3.28 kPa and 171.50 dB/m, respectively. The ranges of LSM and CAP values in our preschool children aged 5 years were 2.00–4.78 kPa and 154.21–214.53 dB/m, respectively.

The FibroScan probe types include S type (S1 and S2), M type and XL type according to the different detection populations. Previous studies and the manufacturer have suggested that the S type probe should be used in preschool and primary school children, whose thoracic circumferences are less than 75 cm [19, 24]. However, it is worth mentioning that the studies that have evaluated the usefulness of TE in children [11, 12] so far have largely been performed in school-age children, using the M(adult) probe [13]. Pradhan et al. found that the S2 probe overestimates the stage of fibrosis compared with the M probe in 10% of patients by evaluating 59 subjects with a thoracic perimeter ≤75 cm, and they believed that the FibroScan M probe should remain the preferred tool for LSM determination in small adults with chronic liver disease [11]. Our study also used the M probe to complete all evaluation in the participating healthy checkup. The total success rate reported in our study was 96.5% which was similar to published adult data [19, 25], but clearly higher than those reported in the pediatric studies by Engelmann et al [21] and Cho et al. [15] The high success rate in our study could be explained by several reasons, such as the parents’ education levels, the small age range and the good degree of cooperation from children and their families. Therefore, if one health center could not afford both the M probe and the S probe considering the total cost, our study results showed that the M probe was also appropriate for detection in these children with correct operation and assurance of accurate results.

In our study, the potential influence of gender on LSM and CAP values using FibroScan was also investigated. The results indicated that the LSM and CAP values did not differ according to gender, which is the same result as reported in other similar studies [19, 20, 26, 27]. Interestingly, there was no correlation between LSM values and all of various demographic, anthropometric, and clinical factors in our anticipating children. However, the situation is different in CAP values. In the univariate analyses of our study, the CAP values were significantly positively correlated with anthropometric measurements (body weight, BMI, waist circumference, fat mass and the percent of body fat), which indicated that CAP values may become a useful tool to assess hepatic fat content and remins doctors to pay attention to apparently healthy children without evidence of sonographic or laboratory abnormalities.

There are some limitations in this prospective study. First, this was a single-center study that included only a sample of preschool children aged 5 years in apparently good health. Further multi-center large study with large sample preschool children are needed, which will validate the normal ranges of LSM and CAP values and consider some other factors. Second, our study was focused on apparently healthy children who did not undergo liver ultrasound and liver biopsies, although liver ultrasound was not recommended for the diagnosis of fatty liver in children considering its lack of sensitivity and specificity, and liver biopsies are also not appropriate for healthy children. Third, this study only focused on an age level. We should continue to discover the normal range at other age levels in future studies based on this ongoing perspective birth cohort.

## Conclusions

In conclusion, these promising results supported FibroScan epuipped with M probe can be used to measure LSM/CAP values in the preschool children aged 5 years. The success rate was similar to that in adult. This study also determined the normal ranges of LSM and CAP values in Chinese 5-year-old children which are different from these in adults. CAP values may become a useful tool to access hepatic fat content in children. Further follow-up with this Shanghai Prenatal Cohort and further study with other large sample sizes are needed to validate the normal ranges of LSM and CAP values at different age levels and whether LSM and CAP can identify those children who are at a higher risk of subsequently developing NAFLD.

## Abbreviations

AKP: Alkaline phosphatase
ALT: Alanine amino transferase
BMI: Body mass index
CAP: Controlled attenuation parameter
dB/m: decibel/meter
GGT: Gamma-glutamyl transferase
HDL-C: High-density lipoprotein cholesterol
IQR: Interquartile range
kPa: kilopascal
LDL-C: Low-density lipoprotein cholesterol
LSM: Liver stiffness measurement
SD: Standard deviations
TE: Transient elastography
TG: Triglyceride
